# Diagnosis and typing of systemic amyloidosis: The role of abdominal fat pad fine needle aspiration biopsy

**DOI:** 10.4103/1742-6413.58950

**Published:** 2010-01-15

**Authors:** Ruba A Halloush, Elena Lavrovskaya, Dina R Mody, Donna Lager, Luan Truong

**Affiliations:** 1 Department of Pathology, The Methodist Hospital, Houston TX; 2 Ameripath South Texas, San Antonio, TX; 3 The Methodist Hospital Research Institute, Houston, TX; 4 Department of Pathology, Mayo Clinic, Rochester, MN

**Keywords:** Abdominal fat pad fine needle aspiration, Congo red stain, systemic amyloidosis, typing of systemic amyloidosis

## Abstract

**Introduction:**

Systemic amyloidosis (SA) has a broad nonspecific clinical presentation. Its diagnosis depends on identifying amyloid in tissues. Abdominal fat pad fine needle aspiration (FPFNA) has been suggested as a sensitive and specific test for diagnosing SA.

**Materials and Methods:**

Thirty-nine FPFNA from 38 patients (16 women and 20 men, age range 40–88 years) during a 15-year period were reviewed. Smears and cell blocks were stained with Congo red (CR). A panel of antibodies (serum amyloid protein, serum amyloid A, albumin, transthyretin, kappa light chain and lambda light chain) was used on six cell blocks from five patients. The FNA findings were correlated with clinical and histological follow-up.

**Results:**

FPFNAs were positive, confirmed by CR in 5/39 (13%), suspicious in 1/39 (3%), negative in 28/39 (72%), and insufficient for diagnosis in 5/39 (13%) of cases. In all the positive cases, SA was confirmed within 2–16 weeks. Among the 28 negative cases, SA was diagnosed in 21, the rest were lost to follow-up. Among the insufficient cases, SA was diagnosed in four and one was lost to follow-up. Specificity was 100%, whereas sensitivity was 19%. SA typing using cell block sections was successful in three, un-interpretable in one, and negative in two cases.

**Conclusion:**

FPFNA for SA is not as good as previously reported. This may be due to different practice setting, level of experience, diagnostic technique, or absence of abdominal soft tissue involvement. A negative result of FPFNA does not exclude SA. Immune phenotyping of amyloid is possible on cell block.

## INTRODUCTION

Amyloid is an insoluble proteinaceous substance, which arranges in beta-pleated sheets and appear as nonbranching linear fibrils under electron microscopy.[1] Amyloidosis represents a spectrum of diseases that results from deposition of amyloid in extracellular matrix, leading to disruption of normal function and a broad but nonspecific clinical manifestation. Up to 24 different types of amyloid precursor proteins have been described, including immunoglobulins, apolipoproteins, proteohormones, transport proteins, and others.[2,3] Amyloid deposits can occur in any organ and may be local or generalized. The localized form of amyloidosis has a better prognosis compared to systemic disease.[4] Amyloid deposits may lead to a wide variety of clinical syndromes, with a wide range of nonspecific symptoms that makes a rapid clinical diagnosis difficult. Adequate treatment of amyloidosis requires not only pathomorphological confirmation of the presence of amyloid, but often its biochemical characterization.

The diagnosis of systemic amyloidosis (SA) requires histological demonstration of amyloid deposition. Amyloid appears as an amorphous, eosinophilic substance that stains pink with the Congo red stain, and displays characteristic apple-green birefringence by polarized microscopy. In the past, rectal and gingival biopsies were considered the gold standard for the diagnosis of amyloidosis and confirmation of the clinical suspicion.[5]

In 1973, Westermark and Stenkvist introduced abdominal fat pad fine needle aspiration (FPFNA) as an alternative method to tissue biopsy to diagnose amyloidosis.[6]

Since then FPFNA has become the preferred diagnostic choice due to its simplicity, low cost and lack of significant complications, with good reported sensitivity and specificity.[7,8] With advanced understanding of the nature and pathophysiology of SA, specific typing of the deposited amyloid protein has become an important factor in treatment and prognostication; however, the utility of FPFNA in this aspect has not been explored.[4]

In this study we reviewed the FPFNAs performed on patients suspected of having SA, with special emphasis on cytologic features, diagnostic utility, and clinicopathologic correlation. The possibility to further subtype the amyloid protein using the cytology material was also evaluated.

## MATERIALS AND METHODS

### Materials

Thirty nine FPFNAs from 38 patients, obtained during a 15-year period (1992–2007) were retrieved from the cytopathology files of the Methodist Hospital, a large tertiary hospital in Houston, Texas. There were 19 women and 19 men (age range: 40–88 years, average: 67 years). Clinical and histological follow-up, including tissue biopsies, for each patient was correlated with the FPFNA findings. Only light microscopy was used to examine the material obtained by FPFNAs. Electron microscopy was not used to examine these materials.

### Methods

In each case, the FNA was performed by a pathologist using a 21–23-gauge needle attached to a 10-ml syringe. An average of five passes was done, and adequacy was evaluated visually by inspecting the specimen for the presence of fat droplets or fragments. Smears were prepared on frosted slides, which retain fatty tissue better than regular slides and prevent loss during staining. The needles were rinsed in CytoLyt® or the tissue culture fluid RPMI for cell block preparation. A cell block was prepared in 22 out of 39 cases using thrombin clot technique after spinning the specimen and taking the supernatant floating fatty tissue.

Congo red stain was performed on smears, as well as on 5-mm cell block sections when available, using the Bennholds method and a modified (alkaline) version, which is documented to improve the detection of amyloid. The slides were subjected to light microscopic examination including polarized light for the presence of apple-green birefringence around fat cells or in small vessels' walls.

Immunoperoxidase stains were performed on cell block sections using the following antibodies: Serum amyloid A (Dako, Carpinteria, CA; clone MC-1, 1:1000), serum amyloid P (Biocare, Concord, CA; 1:20), transthyretin (Dako, Carpinteria, CA; polyclonal, 1:1500), kappa light chains (Dako, Carpinteria, CA; polyclonal, 1:6000), lambda light chains (Dako, Carpinteria, CA; polyclonal, 1:2000) and albumin (Dako, Carpinteria, CA; polyclonal, 1:20,000). This panel was chosen based on the most common proteins involved in SA and the availability of commercial antibodies.

Briefly, tissue sections were deparaffinized and hydrated to water. Slides stained for transthyretin were pre-treated in 44% formic acid, and slides stained for SAA and SAP were pre-treated with a proteolytic enzyme for 5 minutes. All stains were performed on the Dako Autostainer (Dako, Carpinteria, CA) using the Envision+ Dual link-HRP detection system and DAB as the chromogen (DAB+ Dakocytomation K3468). Stained slides were rinsed in water, counterstained with hematoxylin, dehydrated in graded alcohols to xylene and coverslipped. The staining reaction was recorded as positive or negative. These studies were performed for six cell blocks from five patients with confirmed diagnoses of amyloidosis.

## RESULTS

### Cytologic findings

Among the 39 FPFNAs, the findings were positive for amyloidosis, confirmed by Congo red stain in 5 (13%), suspicious in 1 (3%), negative in 28 (72%), and inconclusive for diagnosis in 5 (13%) due to insufficient material.

Positive cases demonstrated faintly eosinophilic, glassy homogeneous material along the contour of adipocytes in smears [Figure 1a]. The same type of material was also noted in the cell block sections, in which cases it was noted not only around adipocytes but also focally in the wall of small blood vessels [Figure 2a]. The Congo red stain highlighted this material in each case in both smears and cell block sections, which also showed apple green birefringence by polarized microscopy [Figures 1b and 2b–d]. This type of material was differentiated from fibrin or hyalinized connective tissue frequently seen in the cell block sections by its characteristic location, a glassier and less eosinophic appearance, and Congo red stain positivity [Figure 2b]. In each of the positive cases, both smears and cell blocks were available and amyloid was noted in both types of tissue preparations.

**Figure 1a:**
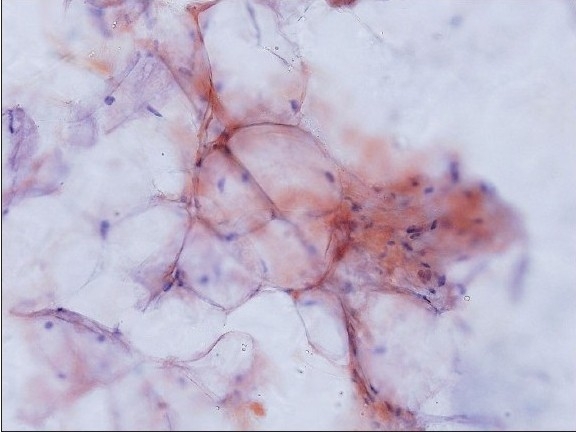
Amyloid on smears: Amyloid appearing as light pink material deposited along the contours of adipocytes (Congo red stain, original magnification × 200)

**Figure 1b:**
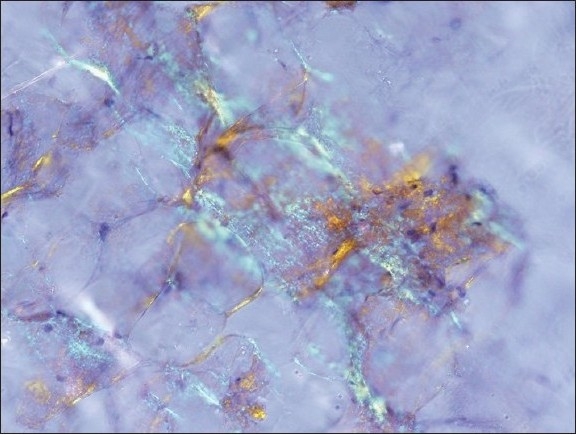
The corresponding polarized light appearance of amyloid with focal apple green birefringence (Original magnification, ×200)

**Figure 2a:**
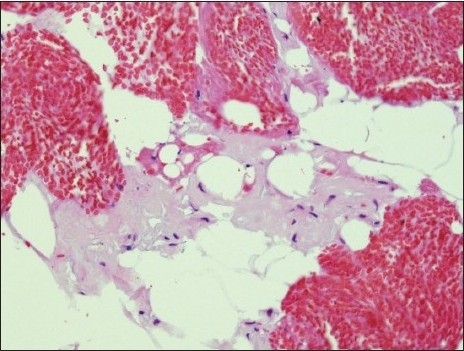
Amyloid on cell blocks: Amyloid appears as glassy faintly esoninophilicmaterial on hematoxylin-eosin stain, deposited around adipocytes (H and E, original magnification × 200)

**Figure 2b:**
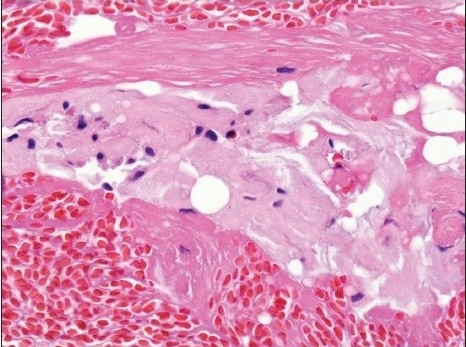
Amyloid sandwiched between two layers of fibrin. Amyloid has an amorphous pink appearance, compared to the deeply pink-red wavy appearance of fibrin (H and E, original magnification × 400)

**Figure 2c:**
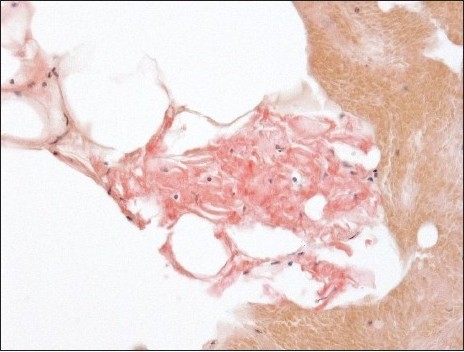
Amyloid appears as pink material deposited between adipocytes (Congo red stain, original magnification × 400).

**Figure 2d:**
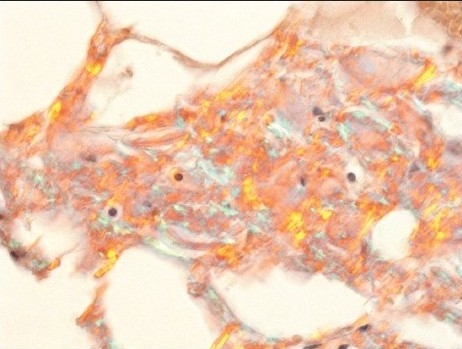
Amyloid displaying focal apple green birefringence under polarized light (C) (Original magnification, × 400)

In the suspicious case, there was deposition of Congophilic material with the Congo red stain; however polarized microscopic examination was equivocal. In the negative cases, amyloid material was not seen in the smears as well as the cellblock, which were available in 19 cases.

The insufficient cases did not have enough adipose tissue and no blood vessels for staining. Cell block preparation attempted on two cases did not yield diagnostic material either.

### Clinicopathologic correlation

Among the 38 patients, the final diagnosis of SA was confirmed by tissue biopsies and or the presence of a monoclonal light chain in 30. The types of amyloidosis included light chain (AL) type in 23 patients, hereditary type in two patients, and remained unclassified in five patients. Chronic diseases known to be associated with SA (secondary, AA type of SA) was not noted in the patients of the last group.

In each of the cases with positive FPFNA, SA was confirmed within 2–16 weeks by follow-up tissue biopsies. All these patients had AL type of amyloidosis. The patient whose FPFNA was interpreted as suspicious for amyloidosis, had a history of multiple myeloma, but amyloidosis was not detected by additional studies. Among 28 patients with a negative FPFNA, SA was finally diagnosed in 21 by tissue biopsies, with the other seven patients being lost to follow-up. The tissue biopsies from which the diagnosis of amyloidosis was made included: kidney (7), myocardium (2), colorectal (5), bone marrow (4), oral mucosa (2) and nerve (1). The types of amyloidosis in these patients included AL type (16), hereditary type (2), and undetermined type (3). Among the five patients with insufficient FPFNA, SA was diagnosed in four by biopsies, and one was lost to follow-up. The types of amyloidosis in these patients included AL type (2) and undetermined type (2).

### Typing of amyloidosis

Amyloid typing was done on six cell blocks from five patients with aconfirmed diagnosis of SA by tissue biopsies. Amyloid was present in at least one level of tissue section from five of these cell blocks. One patient had uninterpretable results the first time so another FPFNA was done. SA typing was successful in three out of six cell blocks, uninterpretable in one due to scant tissue remaining in the cell block, and negative for all the antibodies in two cases [Table 1]. Among the cases that were successfully typed, AL amyloidosis was found in two (one kappa and one lambda light chain, and monoclonal gammopathy was diagnosed later in both patients); the third case was positive for transthyretin with traces of kappa light chain and serum amyloid protein, and a subsequent endomyocardial biopsy confirmed the presence of amyloid. The patient with uninterpretable results had an endomyocardial biopsy that showed amyloid deposition. This was the same patient who had another specimen that was negative for all the antibodies. In the other case that was negative for all tested antibodies, the patient had a kidney biopsy that showed light chain deposition.

**Table 1:** Immunohistochemical Typing of amyloid in cell block sections

*Cases*	*SAP*	*SAA*	*Transthyretin*	*Kappa*	*Lambda*	*Albumin*	*Final typing*
1	Trace	−	+	Trace	−	−	Transthyretin
2	−	−	−	+	−	−	AL
3	+	−	−	−	+	−	AL
4*	Uninterpretable	−	−	Trace	Uninterpretable	−	Transthyretin
5*	−	−	−	−	−	−	Transthyretin
6	−	−	−	−	−	−	AL

* Same patient; SAP = Serum amyloid P; SAA= Serum amyloid A; AL = Light chain type of amyloidosis

### Diagnostic values

The specificity of FPFNA for detecting amyloid was 100%, whereas sensitivity was 19%, taking in consideration the fact that only light micropscopy was used for diagnostic purpose. Positive predictive value was 100% and negative predictive value was 25%.

## DISCUSSION

SA is a heterogeneous group of disorders that results from extracellular deposition of insoluble protein fibrils. The clinical manifestations are largely variable depending on the organs involved and the extent of resultant dysfunction.

Clinical suspicion of SA is supported by detecting the presence of amyloid in tissue, combined with Congo red staining and polarized microscopy. Misdiagnosis may have grave consequences for the patient since treatment, depending on the type of amyloidosis, may include bone marrow transplantation, liver transplantation or chemotherapy.[9]

Fine needle aspiration of abdominal fat is a commonly used method for detecting amyloid. Sensitivity has been reported to range from 55–82% with excellent specificity (over 90%).[10–12] Our study showed a similar specificity and 100% positive predictive value; however, the sensitivity was low at 19%. It should be emphasized that only light microscopy was used for diagnostic purpose. Electron microscopy, which may improve the diagnostic yield, was not used in this study. Nevertheless, a sensitivity of 22%, comparable to our study, was also recently reported.[10] This low specificity could be related to several considerations. First is the possible impact of the size of the needles used for aspiration. In the current study, 21–23 gauge needles were used for aspiration, with an average of five passes. The needle sizes used in previous studies were either larger (18 gauge)[11,13] or of the same sizes as in the current study.[10] Indeed, 22-gauge needles were used in the landmark study by Westermark and Stenkvist, which has popularized the utility of fat pad aspiration for the diagnosis of amyloidosis.[6] It is interesting to note that although the utilized needle sizes are quite variable and can be “large”, the somewhat misleading term “fine needle aspiration of the abdominal fat” remains used for most of these studies.[6,10–13] The diagnostic yield may vary according to the utilized needle sizes. Indeed, MA Ansari-Lari *et al*. reported a sensitivity of around 22%, which is comparable to our result (19%), and 22-gauge needles were used in this study.[10] On the other hand, diagnostic sensitivity ranging from 55–82% was reported in other studies, in some of which 21–22 gauge needles were indeed used.[6] The collective data indicate that 21–23 gauge needles are used in most studies. They also suggest that needles of these sizes are still potentially yield good diagnostic sensitivity, and that the low sensitivity in some of these studies including the current one may not be entirely due to the relatively small sizes of the utilized needles. Another possible explanation is the strict criteria we set for a positive result of Congo redstain which calls for an apple-green birefringence, level of experience and familiarity ininterpreting the Congo red stain, and technical problems. The correct interpretation of the Congo red stain requires experience and is somewhat subjective. van Gameren, in a study of FPFNA, recommended routine independent observation of the Congo red-stained sections for increasing sensitivity.[14] False negative results may also be related to variable or pale staining. Another consideration is the preferential deposition in terms of organ involvement of amyloid depending on its subtype. For example, transthyretin type of amyloidosis has predilection to deposit in the heart; whereas cutaneous deposition is sporadic even in established phase for several other types of hereditary amyloidosis.[15] The observations from this current study, however, do not add significant insight into this matter. This limitation chiefly reflects the observation that most cases of amyloidosis are of AL type, and the amyloidosis type was not determined in several cases. Specifically, chronic inflammatory conditions known to be associated with secondary (AA) type of SA were not obvious in any of these patients. Within the patients with AL amyloidosis, both positive and negative FPFNAs were noted. It is conceivable that the amount of subcutaneous amyloid deposit, which would reflect the duration of SA, may be another determinant of the diagnostic yield. Unfortunately, in most of our cases the duration of amyloidosis is not known, thus precluding a deduction on whether the diagnostic yield of FPFNA would be related to early or advanced amyloidosis. The scant amount of tissue on cell blocks or smears that are available for evaluation is another factor. Use of high-quality polarizing instruments, maximal light intensity and appropriate section thickness for cell block sections (# 10 microns) are all pre-requisite for an accurate diagnosis. Another issue of practical importance is specimen adequacy. As shown by Shidham *et al.*, electron microscopy helps detect amyloidosis in the FPFNA in several cases when light microscopy including the Congo red stain is not diagnostic.[16] Tissue was not submitted to electron microscopic examination in our study, leaving unanswered the question whether the diagnostic yield would be better by this technique. Our unsatisfactory rate was 13%. Among the 22 cell block sections, blood vessels were noted in six. In the six cell block sections with amyloid deposition, blood vessels were noted in two cases and amyloid was noted in vascular wall in both. The data from the current study are therefore limited on whether the presence of blood vessel wall in the aspirated material would increase the diagnostic yield and whether their presence would be considered a criterion for specimen adequacy. Some published observations seem to suggest that using larger needle, for example, 18 gauge ones, to obtain more tissue including vessel wall and submitted tissue for more than one diagnostic tests, including electron microscopy would significantly increased the diagnostic yield.[13,16] These interesting observations, however, need further corroboration.

To decrease the unsatisfactory rate, special precaution is needed in during cell block preparation of the FPFNA. The usual technique of cell block preparation in which after high-speed centrifugation, the supernatant will be discarded and the deposit at the bottom of the tube is harvested for cell block preparation would inevitably lead to loss of diagnostic material. This is due to the fact that fatty tissue, with possible amyloid deposition in it, would floats at the top of supernatant after spinning, so care should be taken not to throw the supernatant away, but instead collect the fatty material and use it to prepare the cell block. Alternatively, the specimen may be filtered and all aspirated debris should be subjected to cell block preparation.

It is no longer enough to make the diagnosis of amyloidosis. With increasing knowledge of the nature and pathogenesis of different amyloid forms, and with the development of specific treatment for certain types of SA, it is necessary to know the biochemical nature of the amyloid involved. Treatment depends on amyloid type, the organs affected, and the level of resulting functional impairment. Though there is no cure for amyloidosis, treatment targeting the cells which synthesize the amyloid precursor proteins may limit further amyloid deposition and improve overall function. Thus, in AL amyloidosis, which results from a monoclonal light chain synthesized by a neoplastic clone of plasma cells, the treatment may include chemotherapy and stem cell transplantation. In AA amyloidosis, a condition associated with chronic inflammation or infection, the treatment of choice is controlling the underlying inflammatory/infectious conditions. In familial amyloidosis, which results from a variety of amylodogenic proteins synthesized by the liver, liver transplant supplying normal proteins instead of the mutant ones is indicated.[17] The typing of SA requires a careful clinical evaluation associated with refined immunohistochemical analysis and genetic testing. The typing may include a combination of amino acid sequence analysis and mass spectrometry potentially applicable to routinely processed tissue.[18,19] Immunohistochemical labeling with amyloid-type specific antibodies, enzyme-linked immunosorbent assay and immunoelectron microscopy.[20–23] Although some of these techniques can be applicable to routinely processed tissue, several of them require a technical level not clinically available in all hospitals, or call for a sizable amount of tissue.

We were able to subtype the amyloidosis in three out of six cell blocks. Although only a small number of cases were studied, the results show that typing is possible using cytology material. To our knowledge, this is the first study addressing this issue. The type of amyloid protein and the extent of tissue involvement are important factors affecting the sensitivity of the results. Two of the cases that we couldn't successfully type ultimately showed transthyretin protein type SA. This protein has predilection for myocardial deposition which might explain the false negative results, although one of our positive cases showed transthyretin. The pattern of involvement might also depend on the stage of disease, with more advanced stages involving subcutaneous adipose tissue. The other negative case was finally typed as AL type with localized organ involvement of the kidney.

Another possibility for false negative cases might reflect the limitation of subtyping inherent to the immunostaining method itself.

In conclusion, FPFNA remains the test of choice for detecting amyloid in patients with a suspicious clinical picture. It is an easy and inexpensive method with minimal complications. It is highly specific; however, its sensitivity may be lower than reported depending on the clinical setting, disease prevalence, the utilized diagnostic techniques, and diagnostic expertise. With the increasing demand to subtype the amyloid protein, immunohistochemical markers can be used on cell block sections.

## COMPETING INTEREST STATEMENT BY ALL AUTHORS

No competing interest to declare by any of the authors.

## AUTHORSHIP STATEMENT BY ALL AUTHORS

All authors of this article declare that we qualify for authorship as defined by ICMJE http://www.icmje.org/#author.

Each author has participated sufficiently in the work and take public responsibility for appropriate portions of the content of this article.

Each author acknowledges that this final version was read and approved.

## ETHICS STATEMENT BY ALL AUTHORS

This study was conducted with approval from Institutional Review Board (IRB) (or its equivalent) of all the institutions associated with this study. Authors take responsibility to maintain relevant documentation in this respect.
